# Nanoelectroablation of Murine Tumors Triggers a CD8-Dependent Inhibition of Secondary Tumor Growth

**DOI:** 10.1371/journal.pone.0134364

**Published:** 2015-07-31

**Authors:** Richard Nuccitelli, Jon Casey Berridge, Zachary Mallon, Mark Kreis, Brian Athos, Pamela Nuccitelli

**Affiliations:** BioElectroMed Corp., Burlingame, California, United States of America; Université Libre de Bruxelles, BELGIUM

## Abstract

We have used both a rat orthotopic hepatocellular carcinoma model and a mouse allograft tumor model to study liver tumor ablation with nanosecond pulsed electric fields (nsPEF). We confirm that nsPEF treatment triggers apoptosis in rat liver tumor cells as indicated by the appearance of cleaved caspase 3 and 9 within two hours after treatment. Furthermore we provide evidence that nsPEF treatment leads to the translocation of calreticulin (CRT) to the cell surface which is considered a damage-associated molecular pattern indicative of immunogenic cell death. We provide direct evidence that nanoelectroablation triggers a CD8-dependent inhibition of secondary tumor growth by comparing the growth rate of secondary orthotopic liver tumors in nsPEF-treated rats with that in nsPEF-treated rats depleted of CD8^+^ cytotoxic T-cells. The growth of these secondary tumors was severely inhibited as compared to tumor growth in CD8-depleated rats, with their average size only 3% of the primary tumor size after the same one-week growth period. In contrast, when we depleted CD8^+^ T-cells the second tumor grew more robustly, reaching 54% of the size of the first tumor. In addition, we demonstrate with immunohistochemistry that CD8^+^ T-cells are highly enriched in the secondary tumors exhibiting slow growth. We also showed that vaccinating mice with nsPEF-treated isogenic tumor cells stimulates an immune response that inhibits the growth of secondary tumors in a CD8^+^-dependent manner. We conclude that nanoelectroablation triggers the production of CD8^+^ cytotoxic T-cells resulting in the inhibition of secondary tumor growth.

## Introduction

Hepatocellular carcinoma (HCC) is the third leading cause of cancer-related death worldwide and the ninth leading cause in the US. According to the International agency for Research on Cancer, HCC is the fifth most common cancer in men (523,000 cases per year with 7.9% of all cancer cases) and the seventh most common cancer in women (226,000 cases per year with 6.5% of all cancer cases) [1]. For patients with lesions less than 2 cm in diameter, radiofrequency ablation (RFA) under ultrasound guidance is the recommended treatment [2].

RFA works by heating the tissue to hyperthermic levels for several minutes resulting in necrosis. Two weaknesses of this technique are the lack of a sharp boundary to the ablation zone and poor ablation around vessels and ducts. Since the spread of heat depends on the thermal conductivity of the tissue, it is difficult to control the precise boundary of this ablation zone. In addition, the presence of heat sinks such as large vessels or ducts allows heat to be carried away from the tissue near the vessel and makes it difficult to reach the temperature required to ablate tissues in those regions.

Here we have used a novel, non-thermal tissue ablation modality called nanoelectroablation that avoids the drawbacks of thermal ablation. Nanosecond pulsed electric field (nsPEF) therapy uses ultrashort, high voltage electric pulses that generate transient nanopores in cell and organelle membranes, leading to the initiation of programmed cell death in the exposed cells[3]. Programmed cell death, also known as apoptosis, is the process used by most of the cells in our body to die when they are old or damaged so it normally does not trigger an immune response. However, over the past decade a form of apoptosis that does stimulate an immune response has been characterized and is called immunogenic apoptosis or immunogenic cell death (ICD). ICD is a cell death modality in which a series of damage-associated molecular patterns (DAMPs) are exhibited. The appearance of DAMPs has been shown to be essential for the stimulation of an immune response and general anticancer immunity *in vivo*. Traditionally, this cell death modality has been initiated by certain chemotherapeutic drugs such as anthracyclines, oxaliplatin, and mitoxantrone[4]. A key requirement for ICD is the combined action of reactive oxygen species (ROS) and ER stress[5]. The ER stress mediated by Ca^2+^ release from the ER is thought to lead to another important component of the signaling pathway in ICD, the translocation of calreticulin (CRT) from the ER to the cell surface. This ecto-CRT becomes an additional “eat me” signal and binds to CD91 and CD69 on dendritic cells to promote phagocytosis, facilitating their tumor antigen presentation and incitement of tumor antigen-specific cytotoxic T-cells[6].

The reason for describing these characteristics of immunogenic apoptosis is that the cellular response to nanoelectroablation exhibits many of these same cellular changes. The large electric field used in nanoelectroablation pushes water molecules into lipid membranes in both the plasma membrane and organelle membranes, forming very small pores or water-filled defects about 1 nm wide[7,8]. Ca^2+^ flows through these nanopores down its concentration gradient moving into the cytoplasm through both the plasma membrane and the ER membrane resulting in ER stress. This increase in cytoplasmic Ca^2+^ in turn stimulates ROS production[9,10]. Therefore nanoelectroporation very rapidly triggers the two cellular events that have been shown to stimulate CRT externalization and immunogenic cell death.

Previous studies from our group have suggested that nanoelectroablation might trigger an immune response. We observed that immunocompetent mice in which one allograft tumor had been nanoelectroablated rejected subsequent allograft tumors while immune-compromised mice did not[11]. However, those experiments were conducted with mice that were not isogenic for the tumor line used. Here we use only isogenic cell lines and explore this immune response in more depth. We find that nanoelectroablation of murine tumors leads to apoptosis as characterized by caspase 3 and 9 activation and the initiation of a CD8-dependent inhibition of secondary tumor growth.

## Materials and Methods

### Laboratory Animals and Cell Lines

For the orthotopic liver tumor experiments we used two-month old male Buffalo rats (Charles River, Cambridge, MA) and the isogenic hepatocellular carcinoma cell line, McA-RH7777, so that there would be no natural immune response to the injected tumor cell line. For the vaccination experiments we used both C57BL/6 female mice and B6 albino female mice (Charles River, Cambridge MA) along with the isogenic MCA205 fibrosarcoma cell line. The animals were housed in the BioElectroMed animal facility and received care according to the *Guide for the Care and Use of Animals* of the National Institutes of Health. All animal studies were approved by the BioElectroMed Institutional Animal Care and Use Committee (Assurance No. A4647-01). All surgery and animal injections were performed under isoflurane inhalation aesthesia, and all efforts were made to minimize distress. The McA-RH7777 cell line was obtained from ATCC and was cultured in DMEM (containing 4mM L-glutamine, 4500 mg/L glucose, 1mM sodium pyruvate, and 1500mg/L sodium bicarbonate), with 10% FBS, and 1% Pen/Strep. The MCA205 cell line was obtained from Andrew Weinberg, Providence Portland Medical Center, Portland, Oregon and cultured in the same DMEM medium. Human pancreatic carcinoma BxPC-3 cells were obtained from ATCC and cultured in RPMI 1640 supplemented with 10% FBS and 1% Pen/Strep. Murine squamous cell carcinoma SCC VII/SF cells were obtained from Allan Balmain at UCSF and cultured in Isacove’s DMEM supplemented with 10% FBS, 1% Penn/Strep and 2% L-Glu.

### Tumor cell injection

**Fig 1:**
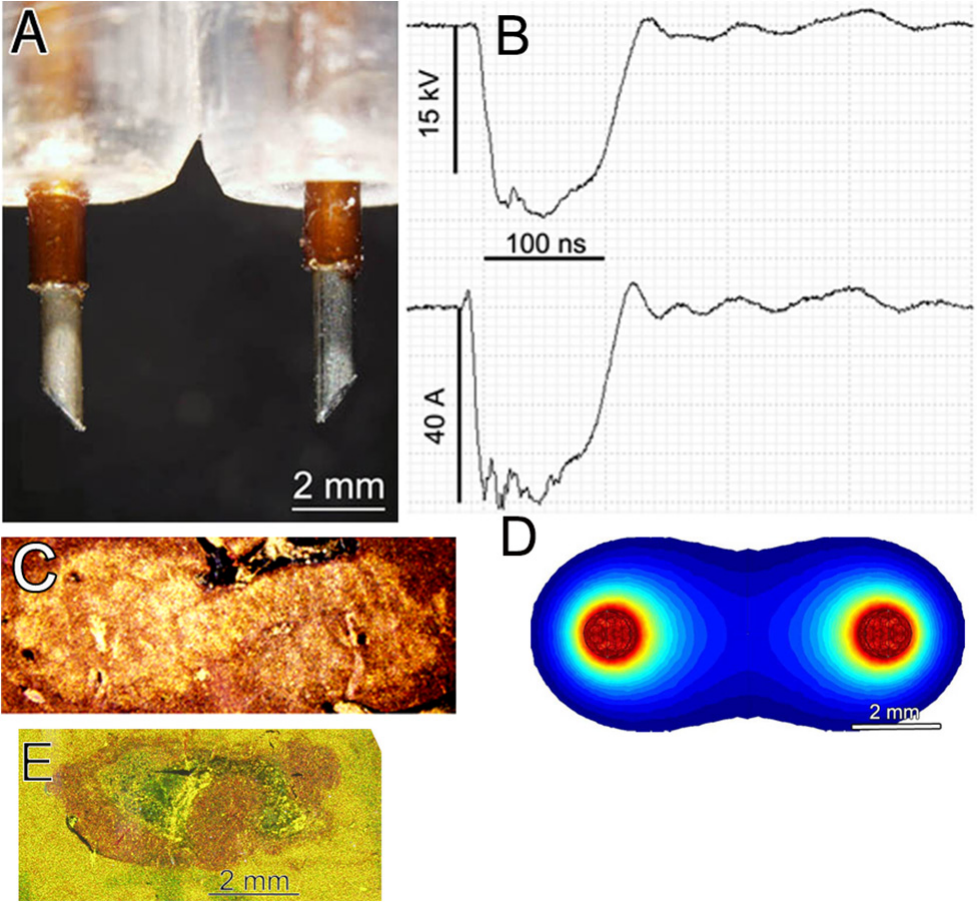
Two-needle electrode used to treat liver tumors. A. Photograph of the electrode. B. Voltage and current wave form delivered by this electrode to the rat liver. C. Scattered light image of 5 μm thick H&E stained section of treated liver 2 h after treatment. D. Model of the predicted electric field generated by these two electrodes when 15 kV is applied between them. The outer boundary represents the 12 kV/cm field line and corresponds well with the boundary of the pyknotic region. E. Scattered light image of an H&E stained section from an untreated liver tumor for comparison with “C”. The difference in light scatter is due to the high concentration of blood cells in the ablation zone.

One million McA-RH7777 cells in 15 μl of HBSS were added to 15 μl MatriGel (Corning) that had been allowed to thaw on ice. The solution was mixed to create a homogenous suspension and kept on ice until the time of injection. A sterile insulin syringe in sealed packaging was also kept on ice until the time of injection. In order to expose the liver for injection, an abdominal incision 3–4 cm long was made on the midline inferior to the xiphoid while the rat was under isoflurane inhalation anesthesia. The 30 μl solution containing 10^6^ cells was injected under the capsule of one liver lobe. We then completed a two-layer closure using absorbable sutures for the peritoneum/muscle layer and wound clips for the skin. One week later the liver was exposed again and photographed and the tumor was treated with the 2-needle electrode delivering 400 pulses (100 ns, 15 kV, 50 A) (Fig 1). After waiting 3 weeks to allow sufficient time for ablation of the primary tumor and the mounting of an immune response, the liver was exposed again and photographed and a second injection of tumor cells was made into another lobe of the liver. One week later the liver was surgically exposed again and photographed to document the size of the second tumor and the rat was euthanized. In some experiments 250 μl of anti-CD8 antibody (Cat #: MCA48EL, BioRad-AbD Serotec) was injected IP 1 day before the second injection of tumor cells to deplete CD8^+^ T-cells. Tumor size was determined by measuring the tumor surface area on the photograph using Image J.

### nsPEF treatment

A 3 mm-long two-needle electrode was used to nanoelectroablate the liver tumors (Fig 1). The needles were 1 mm wide with a 6 mm center-to-center distance. The liver lobe to be treated was supported from below by the sterile plastic handle of a scalpel while the electrode was inserted into the liver to surround the tumor. 400 pulses, 100 ns long and 15 kV in amplitude delivering 50A with a 20 ns rise time were applied. A preliminary study ablating normal liver tissue confirmed that the application of 400 pulses with this electrode was sufficient to completely ablate a dumbbell-shaped liver region 0.5 by 1 cm in size as reported previously[12].

For the vaccination experiments, MCA205 cells were either treated in an electroporation cuvette with 500 pulses (100 ns, 50 kV/cm) to trigger apoptosis or treated with 0.2 mg/ml mitomycin C in DMSO at 37°C for 45 min to trigger necrosis. 200 μl of a cell suspension containing 1.5X10^7^ cells/ml was injected subcutaneously to vaccinate 3 mice for each experimental condition. For the vehicle control we injected the same volume of HBSS containing both Ca^2+^ and Mg^2+^.

### Pulse Generator

All treatments were applied with a 100 ns pulse generator (NanoBlate System, BioElectroMed) that charges a lumped-element pulse-forming network and delivers 15 kV to the electrodes each time a switch (pressure-controlled spark gap) is fired. Pulses were applied at 2–4 pps and the pulse waveform is shown in Fig 1. The typical pulse rise time was 25 ns and a typical pulse delivered 50 A of current (or approximately 0.08 J of energy) into the liver tissue.

### Electric Field Modeling

The two-electrode field modeling results were generated using a 3D model of the geometry. By applying rules of symmetry, only one quadrant of the model was meshed. Sharp joints in the model are avoided to reduce computational instabilities, and a sufficiently fine tetrahedral mesh is used to properly capture field enhancements due to curvatures. Meshing is done with Gmsh: a three-dimensional finite element mesh generator (http://www.geuz.org/gmsh/). Field modeling is done assuming electrostatic conditions. The tissue between the needles is considered homogeneous. Since only electric field strength is of interest, resistivity of the tissue is of no importance. Computation was done with a general environment for the treatment of discrete problems (http://www.geuz.org/getdp/). The underlying equations are those for electrostatics, and have been validated with known analytical solutions. The voltage difference between the electrodes is 15 kV and is assumed to come from an ideal source without impedance (Fig 1D).

### Measuring CD8^+^ T-cell levels

500μl of blood was drawn from the tail vein using heparinized capillary tubes. Blood samples were treated with ACK until all erythrocytes were lysed, spun down, and exposed to 5% PFA. Samples were then washed and the pellet resuspended and deposited on charged slides. Samples were then stained using CD3 (Bio-Rad, cat # MCA772GA) and CD4 (BioLegend, cat. #201511). antibodies, and mounted using DAPI-containing Anti-Fade Mounting Medium. Slides were then imaged using fluorescence microscopy, and images were analyzed by manually counting total number of cells displaying both DAPI and CD3 staining, and subtracting the amount of CD4 stained cells from the total. Both OX-8-injected animals and non-injected animals were used, as well as untreated animals.

### Immunohistochemistry

Liver tissue was fixed in 10% buffered formalin for at least 4 days before embedding in paraffin and preparing 5 μm sections. We probed for cleaved caspase 3 and 9 as well as CD8^+^ T-cells in sections from treated tumors that had been prepared using standard procedures of deparaffinization and antibody labeling. Cleaved caspase 3 and 9 rabbit anti-rat antibodies were obtained from Cell Signalling Technology (cat. #9664P, 9507S). Anti-CD8 antibody was obtained from AbD Serotec (cat. #MCA48EL). Fluorescently labeled secondary goat anti-rabbit antibodies were then used to label the primary antibodies. The calreticulin antibody used to label fixed whole cells was obtained from GeneTex (cat. # GTX62353).

## Results and Discussion

In order to apply nanoelectroablation effectively it is important to identify the precise tissue region that will undergo apoptosis for a given electrode configuration. This region is called the ablation zone. We chose to identify the ablation zone by using one of the earliest indicators of apoptosis, pyknosis. This is a shrinkage of nuclei that occurs by 2 h post treatment. Therefore the ablation zone can be identified by fixing the tissue at this time and sectioning and staining it to identify those cells in the section undergoing pyknosis. We began by treating several rat livers with a two-needle electrode, waiting 2 h before fixing, embedding and sectioning the tissue. When these H&E-stained thin sections are illuminated from the side, we could distinguish the apoptotic region because it scatters light more effectively than the non-apoptotic region. This enhanced light scattering is probably due to the high concentration of red blood cells in the region (Fig 1C). We confirmed that the reflective region coincided with the pyknotic region. For comparison, a section from an untreated tumor is shown in Fig 1E and does not exhibit the enhanced reflectivity.

In order to make the correlation between the ablation zone and the applied electric field, we modeled the E field distribution around the electrodes and we found that the 12 kV/cm boundary coincides quite well with the ablation zone boundary (Fig 1D). Those cells exposed to E fields greater than 12 kV/cm undergo apoptosis and those exposed to smaller fields do not. This creates a very sharp border around the ablation zone. When treating a tumor it is critical that the tumor is localized within this ablation zone to achieve complete ablation.

### Confirming apoptosis initiation by cleaved caspase 3 and 9

**Fig 2:**
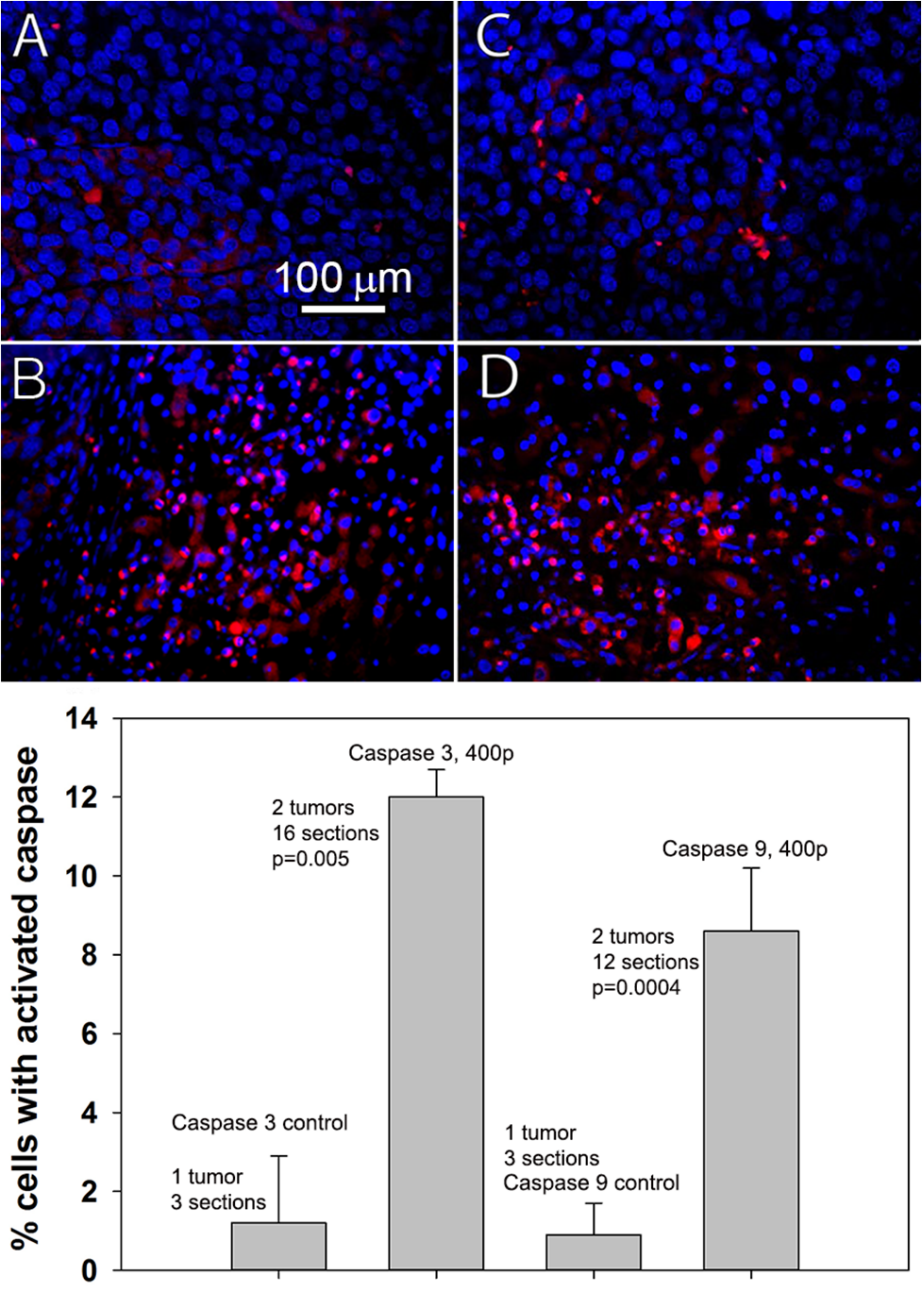
Immunohistochemical labeling of activated caspase 3 and 9. A. Cleaved caspase 3 (red) and DAPI (blue) labeling of thin section of 2-week old untreated control tumor. B. Cleaved caspase 3 (red) and DAPI (blue) labeling of tumor tissue removed 2 h after treatment (400p, 15 kV, 100 ns). C. Cleaved caspase 9 (red) and DAPI (blue) labeling of 2 week old untreated control tumor. D. Cleaved caspase 9 (red) and DAPI (blue) labeling of tumor tissue removed 2 h after nsPEF treatment. Note how the tumor nuclei in B and D have shrunk after treatment, indicating pyknosis. E. Summary of the analysis of 3–7 thin sections taken from one control tumor 14 days after injection and 2 treated tumors fixed 2 h after 400 p, 15 kV. Bars indicate SEM. The difference in activated caspase label between pulsed tissue and controls is highly significant with p = 0.005.

In addition to pyknosis, another hallmark of apoptosis is the activation of caspases 3 and 9 by cleavage of their procaspases. We labeled the cells expressing these activated caspases using immunohistochemistry on thin sections of tissue taken from both control, untreated tumors sampled two weeks after tumor cell injection into the liver, and nanoelectroablated tumors collected 2 h after treatment. Untreated controls showed very low levels of cleaved caspase 3 and 9 (Fig 2A and 2C) while tumor tissue collected 2 h after treatment showed relatively high levels of cleaved caspase 3 and 9 as expected (Fig 2B and 2D). After counting the caspase-positive cells in 3–7 thin sections we found that only 1% of the cells in the control tumors exhibited activated caspase 3 or 9 label compared to 8–12% of the cells in treated tumors. This difference in labeling was highly significant with p = 0.005.

### Effects of nsPEF treatments on liver function

**Fig 3:**
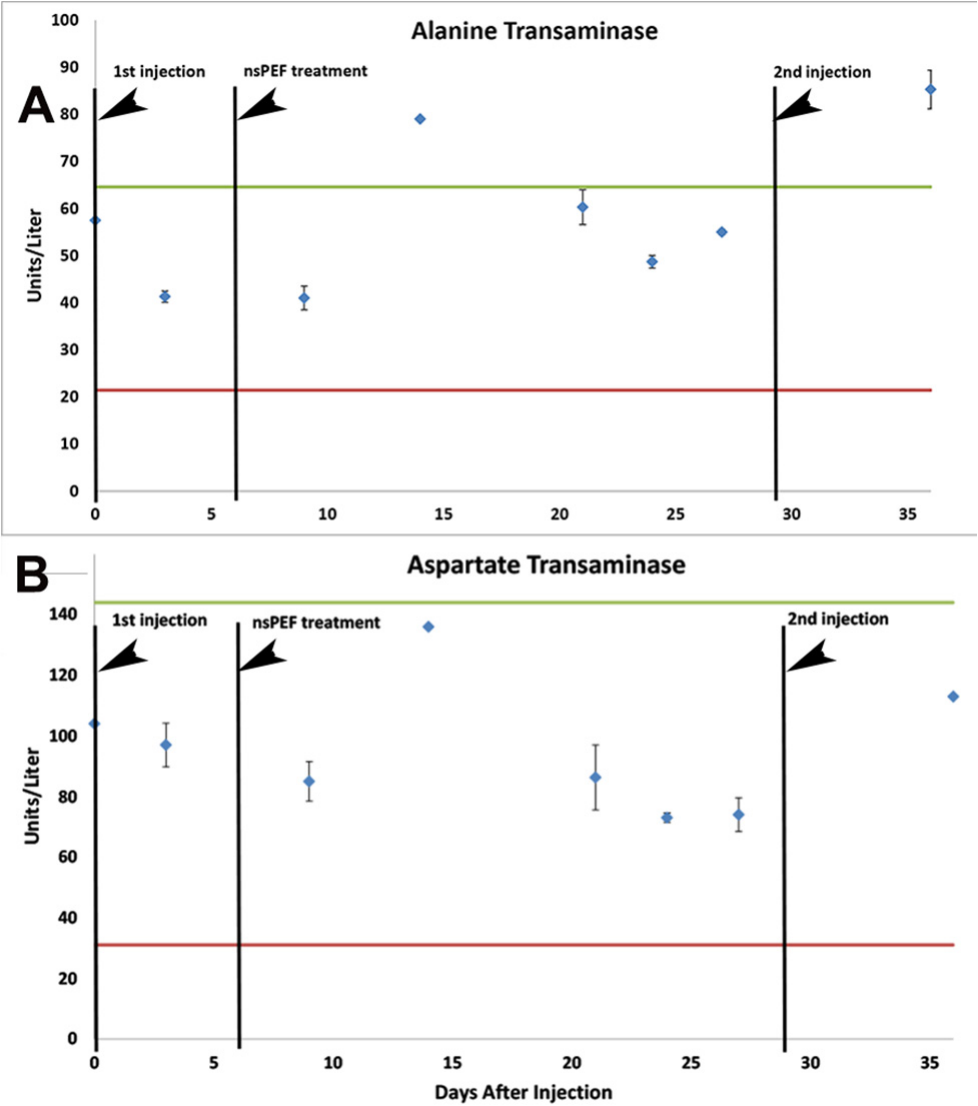
Mean levels of alanine transaminase and aspartate transaminase from 3 Buffalo rats with nsPEF-treated liver tumors. The levels of two liver enzymes were measured. The normal range of these enzymes was determined from blood samples taken from 170 Sprague Dawley rats. The red line indicates the mean level minus 2 standard deviations. The green line indicates the mean level plus 2 standard deviations from the mean.

In order to determine if nanoelectroablating a relatively small region of liver tumor had any effect on the overall liver function, we collected blood samples at various intervals and measured the units per liter of two liver enzymes, alanine transaminase and aspartate transaminase. The average values for these enzymes were obtained by Quality Veterinary Lab (Davis, CA, USA) by collecting blood samples from 170 Sprague Dawley rats. We established the normal range of values for both of these enzymes in Fig 3 to span from a low point given by their mean value minus two standard deviations to a high point given by their mean value plus two standard deviations. Aspartate transaminase levels in all samples collected from rats with treated tumors fell within this normal range. However, alanine transaminase (ALT) levels exceeded this range by about 30% at two time points. The slight increase in ALT 15 days after ablation was transient so is not indicative of permanent liver damage. We conclude that nanoelectroablation of these small tumors does not disrupt overall liver function.

### Calreticulin (CRT) translocation to the cell surface

**Fig 4:**
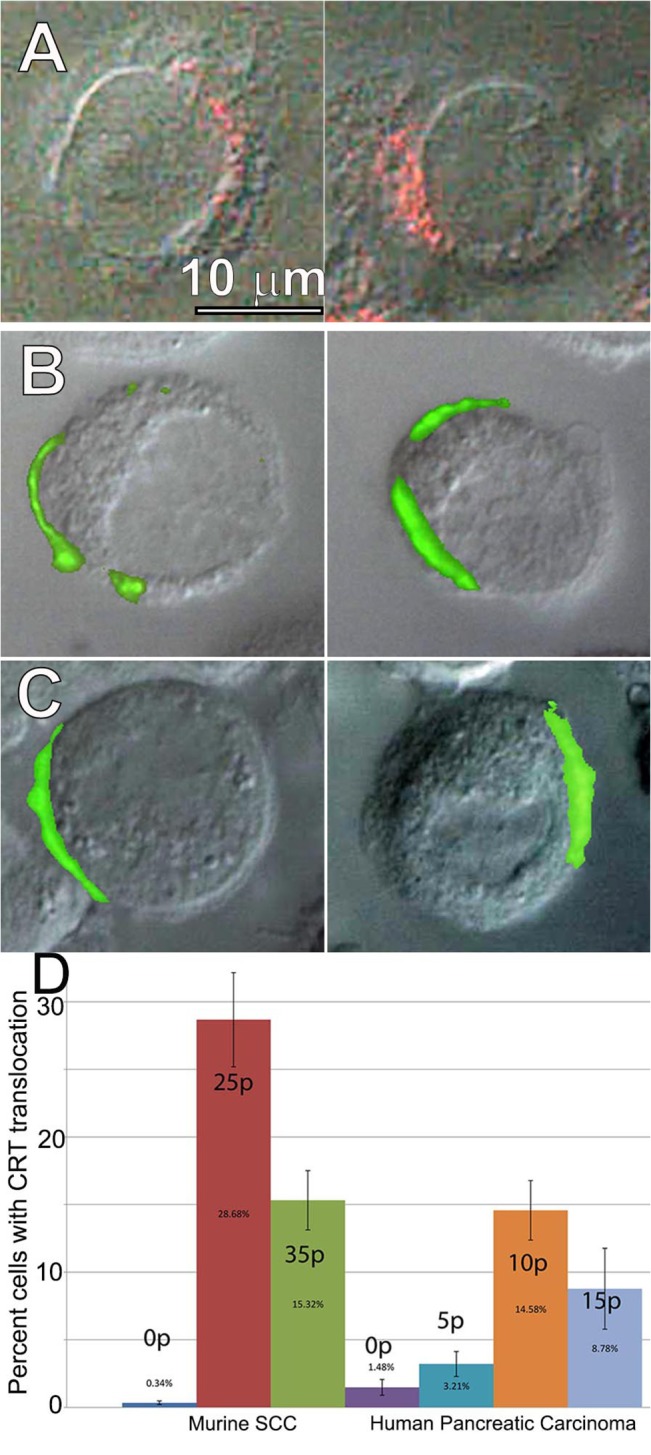
Calreticulin labeling on the surface of cells that had been exposed to nsPEF. **A**. Two typical McA-RH7777 cells labeled with CRT antibody 2h after treating with 25 pulses at 15 kV/cm while plated on an Indium-tin-oxide-coated coverslip that was placed against one electrode in an electroporation cuvette. **B.** Two murine squamous cell carcinoma cells (SCC VII/SF) labeled with CRT antibody 2 h after treating with 15 pulses (25 kV/cm, 100ns). **C.** Two BxPC-3 human pancreatic cancer cells labeled with CRT antibody 2 h after treating with 10 pulses (25 kV/cm, 100 ns). **D.** Percentage of treated cells exhibiting surface CRT 2 h after the indicated number of pulses were applied at 25 kV/cm.

One of the critical DAMPS is the translocation of CRT from the ER to the plasma membrane. We looked for this by labeling nsPEF-treated McA-RH7777 cells with an antibody to CRT and found that CRT translocation could be detected as early as 2 h after nsPEF treatment (Fig 4A). In order to determine if this CRT translocation is a common cellular response to nsPEF treatment, we treated two other cell lines with 25 pulses of 15 kV/cm (Fig 4B and 4C). Both of these cell lines (murine SCC VII/SF cells and human BxPC-3 cells) exhibited CRT translocation and the percentage of cells with surface CRT varied with the number of pulses applied (Fig 4D).

### Nanoelectroablation of the first tumor results in growth inhibition of the second tumor

Since CRT translocation to the cell surface is considered to be one factor that triggers an immune response, we wanted to determine if an immune response is initiated by nanoelectroablation. The classic approach to this question is to determine if secondary tumor growth is inhibited following the ablation of the primary tumor. Chen, et al.[13] have already used a similar orthotopic liver tumor model to show that secondary tumors injected 7 weeks after nanoelectroablation of the primary tumor could not be detected as long as 20 weeks after injection. We took a slightly different approach and looked for these secondary tumors only one week after injection and compared their growth rate with that of the primary tumor prior to ablation in the same rat.

**Fig 5:**
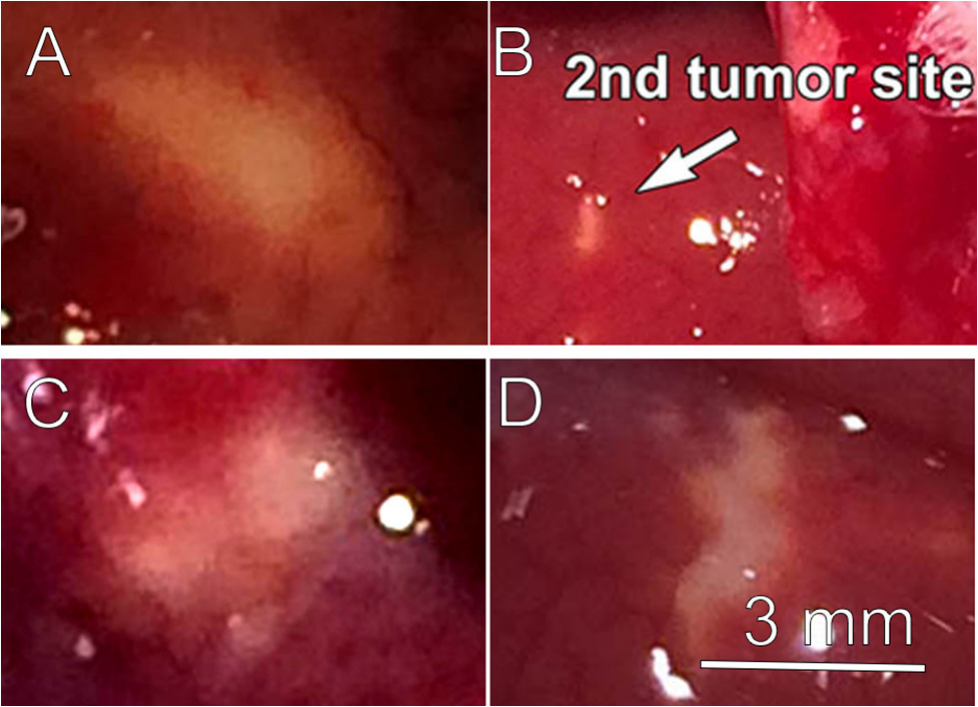
Comparison of secondary tumor growth rates. A. Primary liver tumor 1 wk after injection in rat #30–1. That tumor was treated with 400 pulses, (100 ns, 15 kV) and 3 wk later a second injection of tumor cells was made in another lobe of the same liver; B. Secondary tumor 1 wk after injection in rat #30–1. C. Primary liver tumor 1 wk after injection in rat #36–2. That tumor was treated with 400 pulses (100 ns, 15 kV) and 3 wk later a second injection of tumor cells was made in the same liver in the presence of CD8 antibodies; D. Secondary tumor 1 wk after injection in rat #36–2 after the IP injection of CD8 antibodies. Tumor growth of the second tumor is severely inhibited compared to the first unless CD8 antibodies are present. The scale bar in “D” applies to all images in this figure.

**Fig 6:**
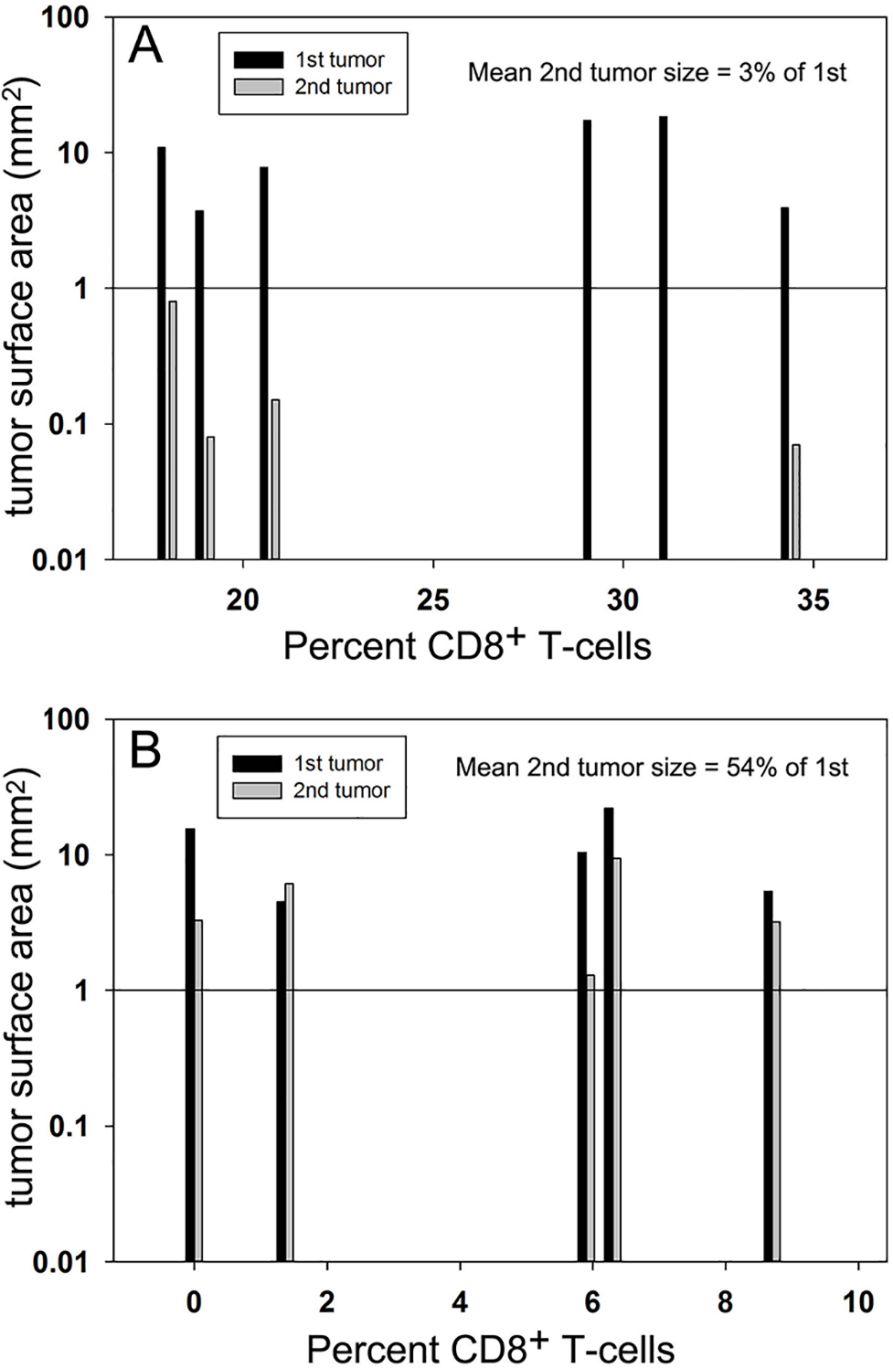
Comparison of 1st and 2^nd^ tumor surface areas in controls (A) and CD8^+^-depleted rats (B). We took blood samples from each rat and counted the number of both CD4^+^ and CD8^+^ cells. The percentage of CD8^+^ cells in this total is plotted for each tumor pair. We plotted the tumor surface area on a log scale to highlight the dramatic increase in the rate of growth of the second tumor when CD8^+^ cells were depleted. In two of the controls, we could not find the second tumor so the mean tumor size was calculated from the four visible second tumors. A Mann-Whitney Rank Sum Test indicates a statistically significant difference between the secondary tumor sizes in these two groups. (P = 0.03).

We surgically exposed the liver and injected one million tumor cells beneath the capsule of one liver lobe in 6 rats. After closing the incision, we waited 1 week before reopening and photographing the tumor which was typically 4–6 mm wide at that time. We then nanoelectroablated the tumor with 400 pulses (100 ns, 15 kV) applied between needles 6 mm apart center-to-center (Fig 1). We closed the incision and then waited three weeks for the treated tumor to be ablated and for an immune response to develop. We then exposed the liver again to allow the second injection of tumor cells beneath the capsule of another liver lobe. The incision was then closed and we waited one week for the second tumor to grow. We then sacrificed the animal and removed the entire liver for photographs and histological analysis (Fig 5). These second tumors failed to grow well and in two instances could not be found at all (Fig 6). When we measured the mean surface area of those tumors that we did find, it was only 3% of the mean area exhibited by the primary tumors after the same 1 week of growth (Fig 6A).

### Depletion of CD8^+^ T-cells reverses the growth inhibition of the second tumor

One characteristic of the adaptive immune response is the generation of CD8^+^ cytotoxic T-cells that specifically kill cells with surface antigens to which they are targeted. A common test for the involvement of CD8^+^ T-cells in an apparent immune response is to use specific antibodies to these cells to deplete them from the animal and determine if this has any effect on the apparent immune response.

We carried out this experiment by first nanoelectroablating primary tumors with the same nsPEF treatment in another group of 5 rats, but 1 day prior to the second injection of tumor cells we performed an intraperitoneal (IP) injection of 250 μg of the anti-rat CD8^+^ monoclonal antibody, OX-8. We collected blood samples before and at least 24 h after injection to confirm the depletion of CD8^+^ cells and included these data in the bar chart. We waited 1 week after injecting the second tumor before sacrificing the rat and processing the liver for immunohistochemistry. The second tumors grew much faster under CD8^+^-depleted conditions with the mean 2^nd^ tumor size equal to 54% of that of the 1^st^ tumor and in one case it was larger than the first tumor (Figs 5 and 6, S1 Fig, S2 Fig).

### Immunohistochemistry detects CD8^+^ T-cells within the tumor

**Fig 7:**
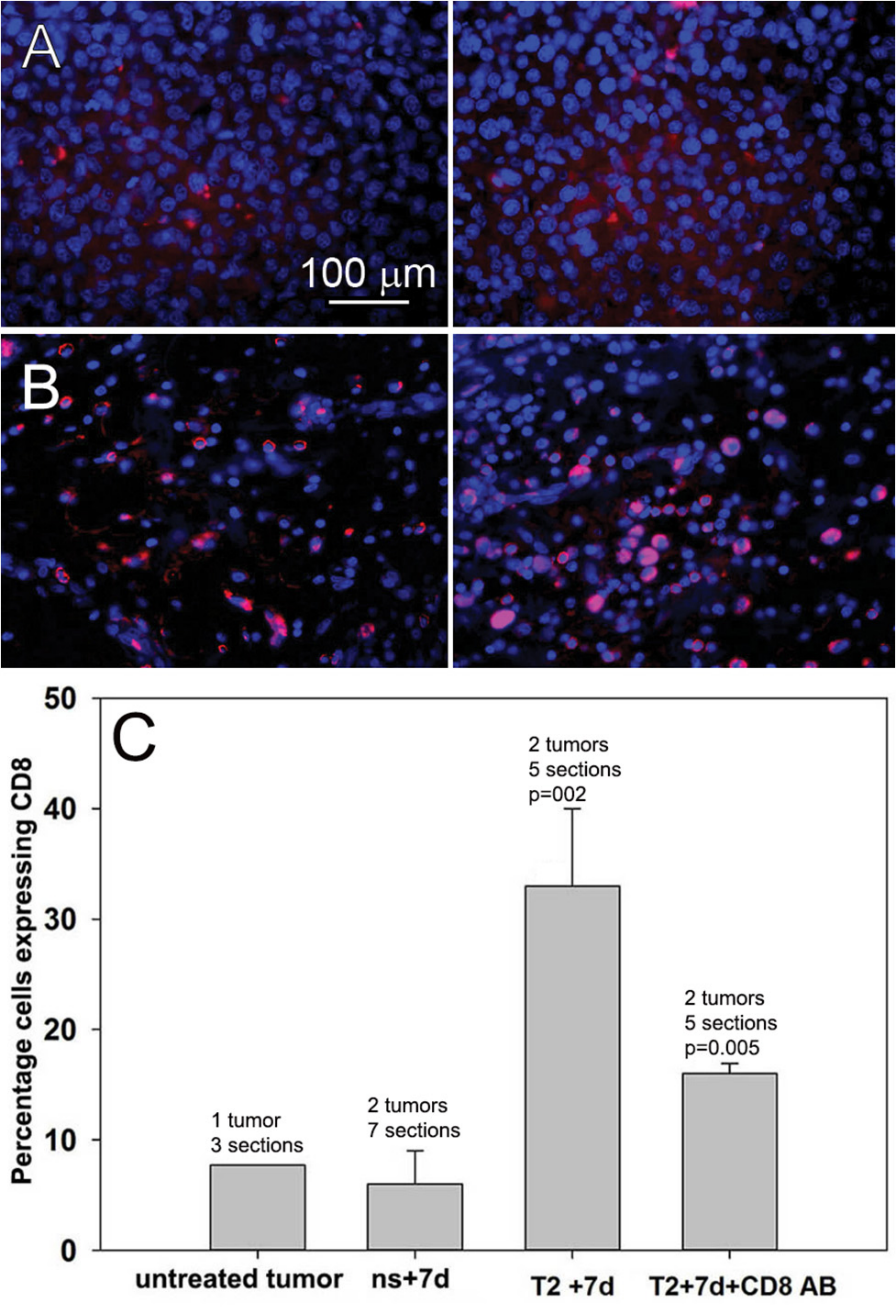
Immunohistochemical labeling of tumor thin sections with CD8 (red) antibodies and DAPI (blue) nuclear stain. A: Two typical sections from an untreated control tumor collected 14 days after injecting tumor cells into the liver; B. Two typical sections of a secondary tumor that was fixed 7 days after it was injected into a liver in which the primary tumor had been nanoelectroablated 3 weeks earlier. This secondary tumor exhibited growth inhibition and a photograph of it can be seen in Fig 5B; C. Percentage of cells expressing CD8 in histological sections of tumors from four different conditions. Each bar represents the mean of measurements taken from 3–7 sections of 2 separate tumors. The untreated tumors were fixed 14 days after injection; “ns+7d” represents tumors fixed 7days after treating with 400 pulses, 15 kV, 100ns; “T2+7d” represents secondary tumors fixed 7 days after injection. CD8 concentration is significantly different from control tumor levels with **p = 0.002; “T2+7d+CD9 AB” represents secondary tumors fixed 7 days after injection with CD8 antibodies injected IP 24 h prior to the tumor cell injection. The CD8 concentration is significantly different from control tumors with *p = 0.005.

In order to directly investigate the presence of CD8^+^ T-cells within the nanoelectroablated tumors, we sectioned fixed tumors and incubated the sections with antibodies to this surface molecule (Fig 7). Untreated control tumors showed sparse labeling with CD8^+^ antibody (Fig 7A). Sections of tumors removed 7 days after nanoelectroablation as well as sections taken from a secondary tumor that exhibited growth inhibition, exhibited 5-fold more cells expressing CD8 (Fig 7B and 7C). We counted the percentage of CD8^+^ cells in 3–7 histological sections from two treated tumors for each of 4 conditions. There was a significant increase in CD8^+^ cells in secondary tumors fixed 7 days after injection compared to the small numbers in untreated tumors or in primary tumors 7 days after treatment. This result supports our hypothesis that CD8^+^ cytotoxic T-cells are involved in inhibiting tumor growth.

### Vaccination with nsPEF-treated tumor cells stimulates an immune response

**Fig 8:**
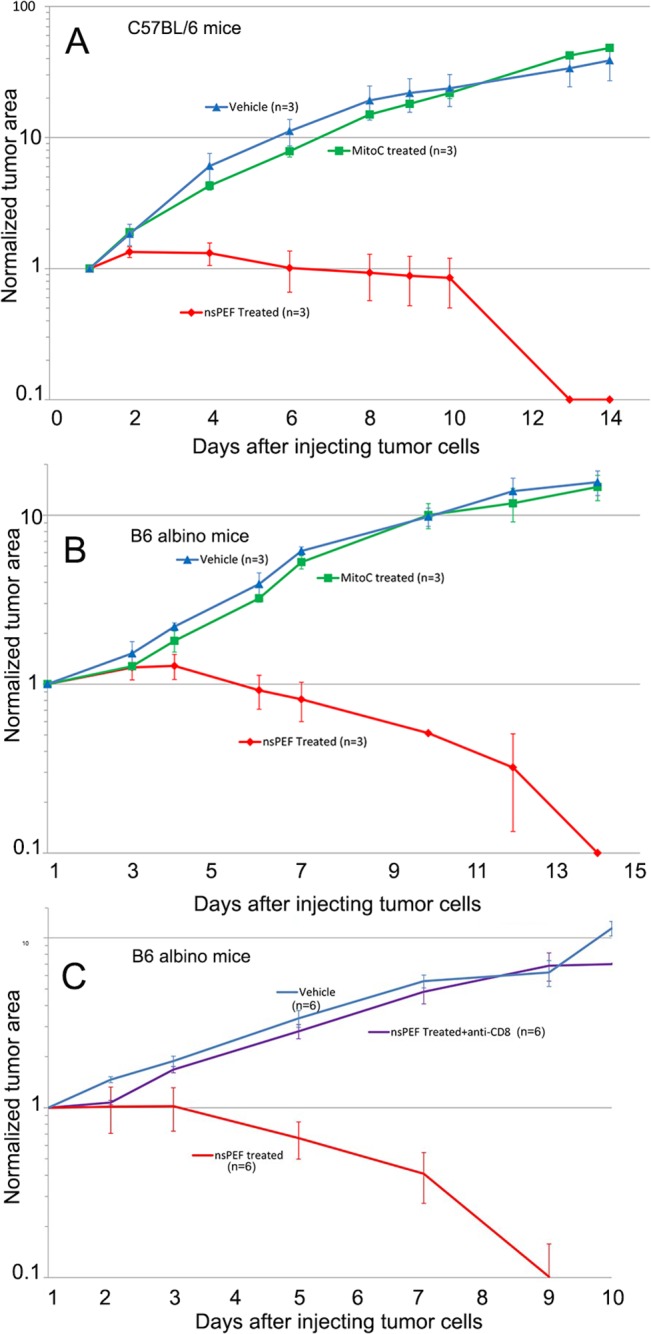
Normalized growth rates of second tumor from MCA205 cells injected 3 weeks after the mouse was vaccinated with either vehicle or MCA205 cells that were treated as indicated. A. Average tumor growth rates in 3 Black C57BL/6 mice vaccinated with saline or mitomycin C-treated cells or nsPEF-treated cells. There is no significant difference between the tumor growth rates in the two controls (p = 0.7). There is a very significant difference between the tumor growth rate in the two controls and that in the mice vaccinated with nsPEF-treated cells (p<0.001). This statistical analysis provided the same conclusion for A-C. B. Average tumor growth rates in 3 B6 albino mice vaccinated with saline or mitomycin C-treated cells or nsPEF-treated cells. C. Average tumor growth rates in 6 B6 albino mice vaccinated with either saline vehicle or nsPEF-treated cells. One of the groups (purple line) was injected with anti-CD8 antibody 24 h prior to injecting the tumor cells. The error bars represent the SEM.

**Fig 9:**
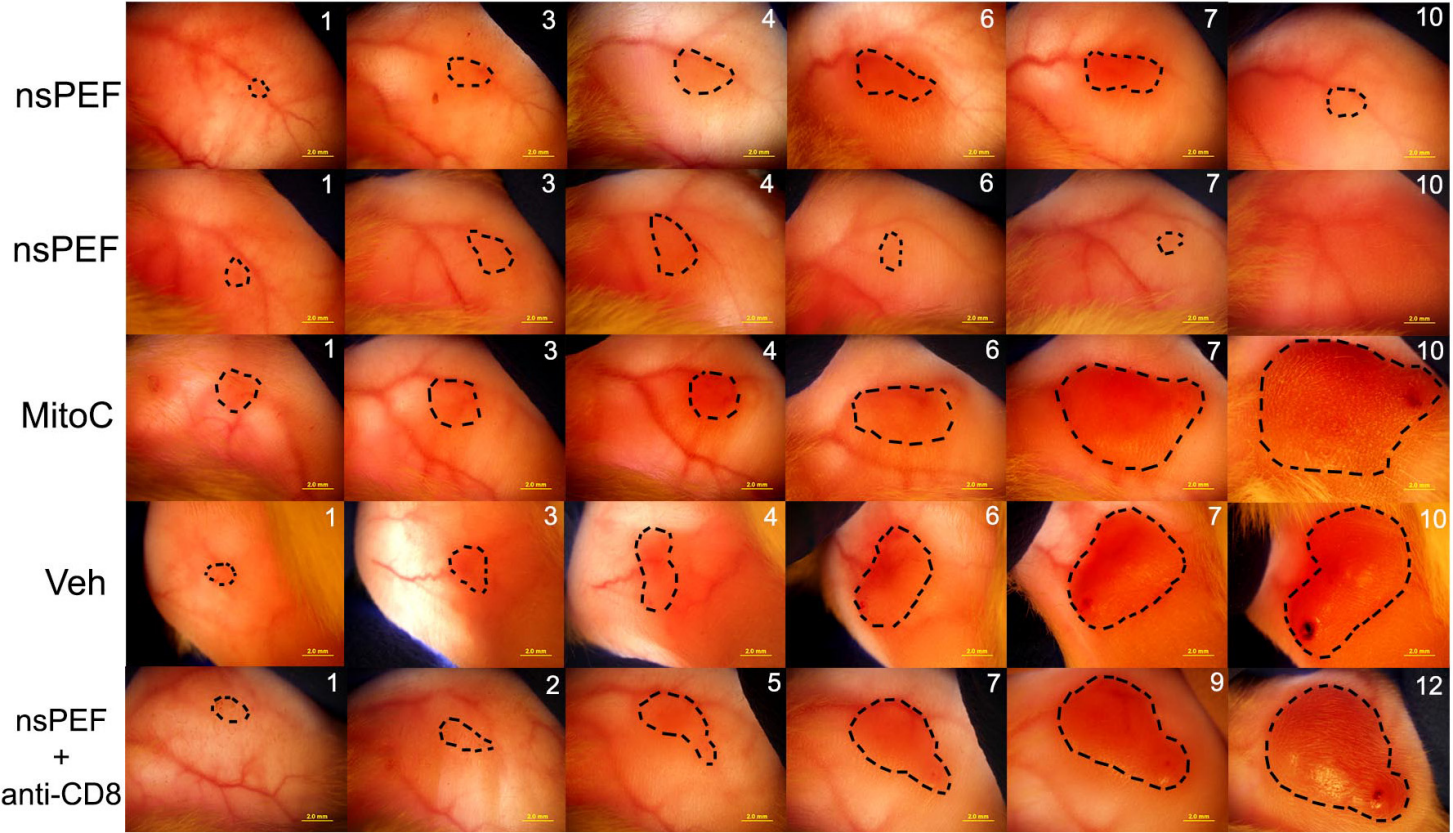
Transillumination images of subdermal MCA205 tumors in isogenic B6 albino mice. Tumors are outlined with dotted lines to aid in visualization. Two top rows show tumors growing from cells injected into mice vaccinated 3 weeks earlier with nsPEF-treated MCA205 cells. The 3^rd^ row down shows a tumor growing from cells injected into a mouse vaccinated with mitomycin C-treated cells. The fourth row down shows a tumor growing from cells injected into a mouse vaccinated with saline vehicle. The bottom row shows a tumor growing from cells injected into a mouse vaccinated with nsPEF-treated cells one day after injecting anti-CD8 antibodies IP.

Our observation that liver tumor nanoelectroablation stimulated a CD8-dependent immune response suggested another experiment. Perhaps treating tumor cells *in vitro* with nsPEF would initiate immunogenic apoptosis in those cells so that injecting them into mice would activate the mouse’s immune system. This “vaccination” with nsPEF-treated cells should also activate the immune system to inhibit secondary tumor growth. We treated the MCA205 fibrosarcoma cell line with nsPEF (50 kV/cm, 100 ns, 500 pulses) in a cuvette and injected 3 million of these cells subcutaneously into syngeneic C57BL/6 or B6 mice. These injected, nsPEF-treated cells did not grow into a tumor. We then waited three weeks for an immune response to develop and injected 1 million healthy MCA205 cells subcutaneously and measured the subsequent tumor size over time. These secondary tumors failed to grow and instead shrunk away until they were no longer visible (Figs 8 and 9). However, secondary tumors in control animals that had been vaccinated with vehicle or mitomycin C-treated cells grew very well. Mitomycin C treatment kills cells by necrosis rather than apoptosis. Since we suspected that the growth inhibition and tumor shrinkage observed in mice vaccinated with nsPEF-treated cells was due to the presence of CD8^+^ T-cells, we conducted another experiment in which we depleted these CD8^+^ cells by injecting anti-CD8 antibodies IP 24 h prior to injecting the secondary tumor. These tumors grew just as fast as the controls (Fig 8C), supporting our hypothesis that an adaptive immune response was responsible for the inhibition of secondary tumor growth.

We provide the first direct evidence of CD8-dependent inhibition of secondary tumor growth following primary tumor nanoelectroablation. This evidence includes: 1) A five-fold increase in the concentration of cytotoxic CD8^+^ T-cells in secondary tumors injected 3 weeks after the primary tumor was nanoelectroablated; 2) The inhibition of secondary tumor growth from tumor cells injected 3 weeks after the primary tumor is nanoelectroablated; 3) The partial reversal of this secondary tumor growth inhibition when prior antibody injection reduces CD8 levels; 4) The ability of injected nsPEF-treated syngeneic tumor cells to stimulate a CD8-dependent inhibition of secondary tumor growth. While these four results are consistent with the initiation of an adaptive immune response, we have yet to show that these CD8^+^ T-cells are specific for the tumor cell antigens.

### How is it that the physical treatment with nsPEF can have the same immunogenic effect as some chemotherapeutic drugs?

What we know of the nanoelectroablation mechanism suggests that the transient E field-induced nanopore generation elicits both ROS and ER stress[9,10,14,15]. The large electric field imposed on the cell by the nsPEF drives water into the lipid membranes to form nanopores that allow Ca^2+^ to flow into the cytoplasm from both outside the cell and from ER stores[16]. The loss of Ca^2+^ stresses the ER and the increase in cytoplasmic Ca^2+^ triggers ROS generation[9]. Therefore, it appears that the physical process of generating transient nanopores in cellular membranes results in the same ROS formation and ER stress previously observed following the application of some chemotherapeutic drug families[17]. This combination of ROS and ER stress was found to lead to calreticulin translocation to the plasma membrane to initiate the immune response[6].

### What are the advantages of nanoelectroablation over other ablation modalities?

Most current ablation modalities apply heat to kill the tumors by hyperthermia. Tissue regions located near blood vessels or ducts resist thermal ablation because the heat is carried away by fluid flow in these vessels. Nanoelectroablation offers a non-thermal, precisely targeted mechanism to trigger immunogenic apoptosis in tumors regardless of vessel or duct locations. The ablation zone has a very sharp outer boundary so that the targeting of tumors is much more accurate than with techniques using hyperthermia. In addition, this ablation modality appears to be effective on all tumor types. It has been used effectively on both allograft and xenograft tumor models[18–21] as well as transgenic murine models[11,22].

Another non-thermal ablation modality that uses pulsed electric fields is called the NanoKnife. It uses 1000-fold longer pulses that result in irreversible electroporation (IRE) that kills cells by necrosis and is not immunogenic. The main advantage of nanoelectroablation over IRE is reduced muscle stimulation in addition to the initiation of an immune response. IRE therapy requires that the patient is paralyzed by muscle blockade but nanoelectroablation does not require that.

### What previous work suggested that nanoelectroablation inhibits secondary tumor growth?

The stimulation of the immune response was first reported using a murine melanoma allograft model system[11] and has also been implicated in experiments using three other model systems[13,23,24]^.^ Our murine melanoma allograft work demonstrated that nanoelectroablation was superior to surgical excision at accelerating secondary tumor rejection in immune-competent mice but not in immunodeficient mice. This suggested enhanced stimulation of a protective immune response by nsPEF treatment and that was further supported by the presence of CD4^+^ T-cells within the treated tumors as well as within untreated tumors in mice with other melanomas that had been treated with nanoelectroablation at least 19 days earlier. Chen, et al. [24] found that macrophage infiltration significantly increased in tumors that had been treated with nsPEF and Yin et al.[23] observed that non-lethal nsPEF treatment increased the rate of macrophage phagocytosis of treated cells and also decreased lung metastasis from the primary tumor. Most recently Chen et al. [13] used another orthotopic rat liver tumor model similar to that described here and observed that in 23 rats the secondary tumor failed to grow after the first was nanoelectroablated. They also observed the presence of granzyme B-secreting cells which could implicate either T-cells and the adaptive immune response or natural killer cells and the innate immune response. Here we directly test for the nsPEF-induced stimulation of the adaptive immune response by observing the effect on secondary tumor growth of specifically depleting CD8^+^ cytotoxic T-cells.

The implications of this immune stimulation are profound as it raises the possibility that nanoelectroablating one tumor could enlist the immune system to attack untreated metastases or even unablated regions of the original tumor if it is slow growing. This immunogenic characteristic of nanoelectroablation may be the most important characteristic of this ablation modality. We also demonstrate that this immunogenic characteristic extends to mice as well. When we vaccinated mice with nsPEF-treated cells, injected tumors shrunk while growing normally in mice that had been vaccinated with vehicle or with necrotic tumor cells. This confirms earlier observations that necrotic cells are unable to stimulate the same immune response triggered by apoptotic cells[25].

### Immunogenic Cell Death in Other Ablation Modalities

Several other ablation modalities have also reported the ability to stimulate the immune system, although none of them directly showed the involvement of CD8^+^ T-cells. Lluis Mir showed that electrochemotherapy (ECT) with bleomycin induces hallmarks of immunogenic cell death [26]. They reported that 8 pulses 100 μs long, 1.3 kV/cm were sufficient to stimulate calreticulin externalization and ATP release but the presence of bleomycin was required for HMGB1 release. Moreover, vaccination with ECT-treated cancer cells protects mice against subsequent challenge with normal cancer cells. Additional ablation modalities eliciting immunogenic cell death include radiation therapy [27], photodynamic therapy [28,29], and microwave thermal ablation[30]. These latter two were both demonstrated to provide protection against subsequent challenge following vaccination. It is not clear what these diverse modalities have in common that allows them all to stimulate immunogenic cell death. ROS stimulation has been observed for electropermeabilization[31], radiation and photodynamic therapy but not yet for microwave thermal ablation, so that may be the common link between these modalities. The main point is that enlisting the immune system to attack metastases provides a very powerful advantage to these ablation techniques over other ablation methods that do not trigger ICD. It remains to be determined which of these ablation modalities triggers the strongest immune response.

## Conclusions

Nanoelectroablation does much more than simply ablate tissue. In both the orthotopic rat liver tumor model system and the mouse fibrosarcoma allograft system, treatment with nsPEF resulted in the inhibition of secondary tumor growth. We provide the first evidence for the involvement of CD8^+^ T-cells in this tumor growth inhibition and provide evidence that nsPEF-treated tumor cells can be used to vaccinate mice against subsequent tumor growth. While similar observations of immunogenic cell death have been observed following electropermeabilization, radiation and photodynamic therapy, evidence for the involvement of cytotoxic CD8^+^ T-cells has not yet been reported in these other ablation modalities

## Supporting Information

S1 Fig Comparison of liver tumor sizes before and after nanoelectroablation of primary liver tumor. Column T1: Six primary liver tumors photographed 1 week after injection. Column T2: Six secondary liver tumors photographed 1 week after injection into the same liver in which corresponding primary tumor (T1) was nanoelectroablated 4 weeks earlier. The ratio of tumor surface areas (T2/T1) is indicated to the right of each pair of images and the mean ratio is 0.03±0.01. The two cases in which no secondary tumor was detected are not included in this average ratio.
(TIF)

S2 Fig Comparison of tumor sizes before and after nanoelectroablation of the primary tumor in a CD8-depleted rat. Column T1: Six primary liver tumors photographed 1 week after injection. Column T2: Six secondary liver tumors photographed 1 week after injection into the same liver in which corresponding primary tumor (T1) was nanoelectroablated 4 weeks earlier. 1 day before injecting the second tumor, CD8 antibody was injected IP to deplete CD8 cells. The ratio of tumor surface areas (T2/T1) is indicated to the right of each pair of images and the mean ratio is 0.54±0.2 (SEM).
(TIF)
