# Supplementary figures and images for: Nanoelectroablation of Murine Tumors Triggers a CD8-Dependent Inhibition of Secondary Tumor Growth
Source: PLoS One. 2015 Jul 31;10(7):e0134364. doi: 10.1371/journal.pone.0134364 (PMC4521782; doi:10.1371/journal.pone.0134364)

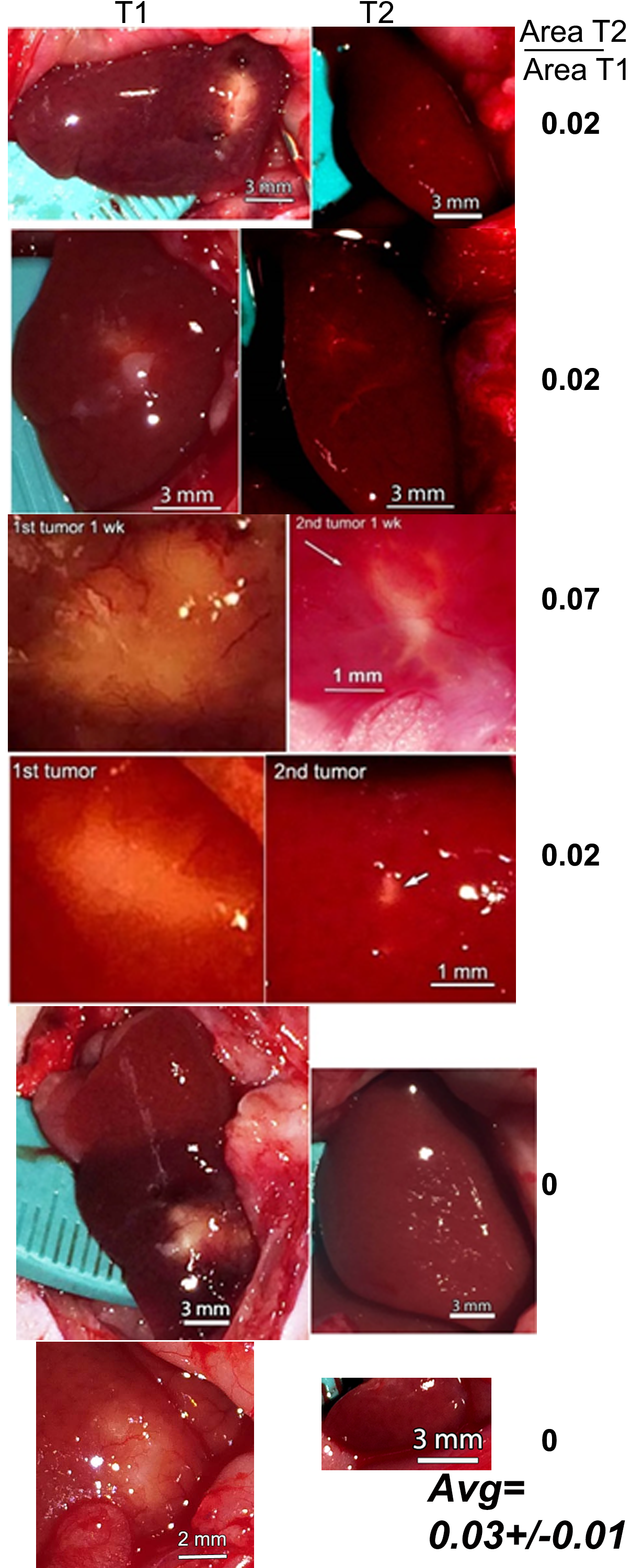

Supplement: S1 Fig — Column T1: Six primary liver tumors photographed 1 week after injection. Column T2: Six secondary liver tumors photographed 1 week after injection into the same liver in which corresponding primary tumor (T1) was nanoelectroablated 4 weeks earlier. The ratio of tumor surface areas (T2/T1) is indicated to the right of each pair of images and the mean ratio is 0.03±0.01. The two cases in which no secondary tumor was detected are not included in this average ratio. (TIF) [file pone.0134364.s001.tif]

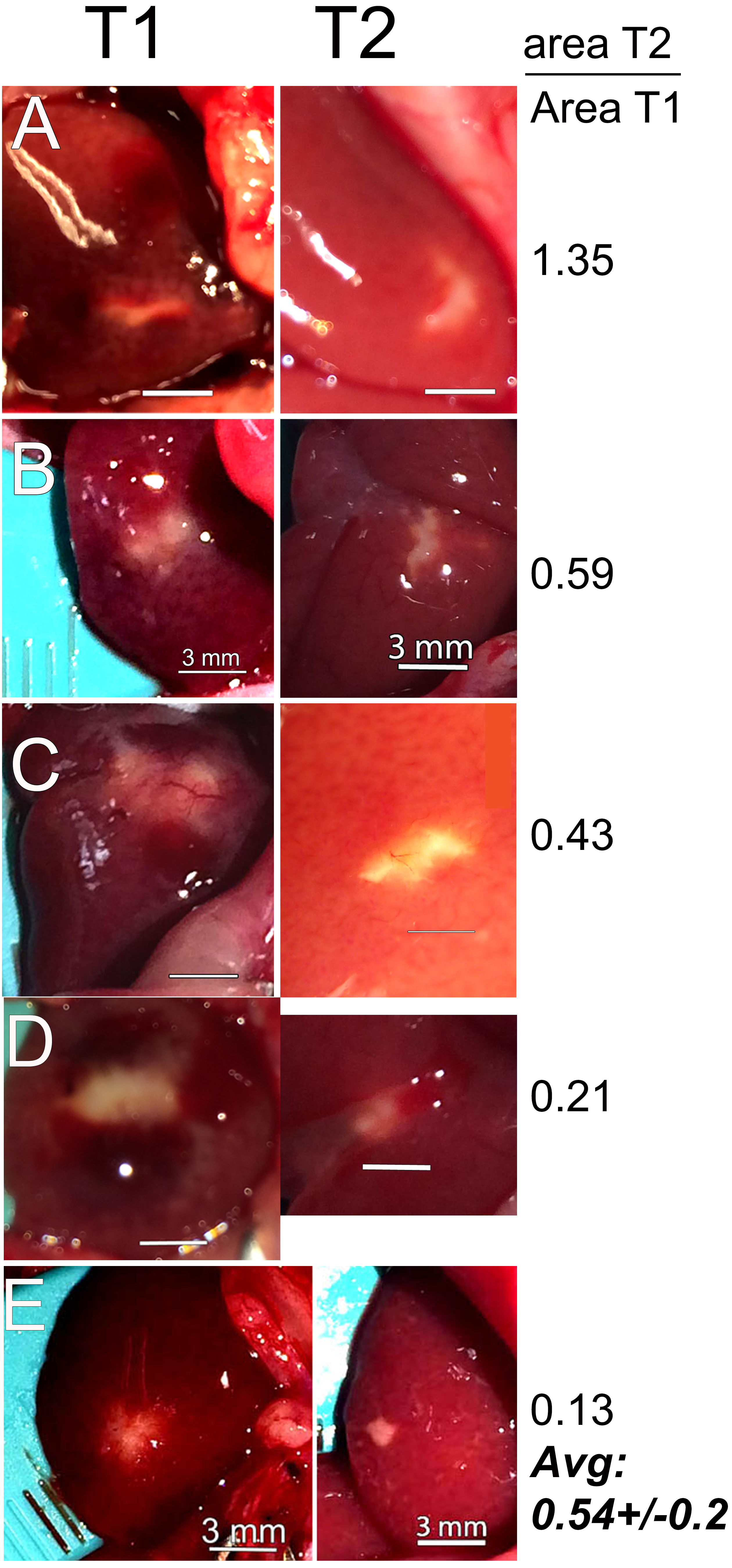

Supplement: S2 Fig — Column T1: Six primary liver tumors photographed 1 week after injection. Column T2: Six secondary liver tumors photographed 1 week after injection into the same liver in which corresponding primary tumor (T1) was nanoelectroablated 4 weeks earlier. 1 day before injecting the second tumor, CD8 antibody was injected IP to deplete CD8 cells. The ratio of tumor surface areas (T2/T1) is indicated to the right of each pair of images and the mean ratio is 0.54±0.2 (SEM). (TIF) [file pone.0134364.s002.tif]
